# Transient Receptor Potential (TRP) Channels: Markers and Therapeutic Targets for Cancer?

**DOI:** 10.3390/biom12040547

**Published:** 2022-04-06

**Authors:** Maria Beatrice Morelli, Consuelo Amantini

**Affiliations:** 1 School of Pharmacy, Section of Experimental Medicine, University of Camerino, 62032 Camerino, Italy; 2 School of Biosciences and Veterinary Medicine, University of Camerino, 62032 Camerino, Italy

This Special Issue in *Biomolecules* explores the roles of Transient Receptor Potential channels (TRPs) in cancer. The main goal is to collect articles that describe recent progress in elucidating the mechanisms by which these channels modulate tumor progression. Despite extensive research efforts, neoplastic diseases remain a leading cause of death and morbidity worldwide, together with the rapid evolution of resistance in tumor cells to the current therapies. Therefore, clinical research must promote innovative strategies to target unexplored cancer-relevant markers such as TRP channels, which could hold promise. Several natural products, such as capsaicinoids, cannabinoids, and terpenes, are known to be TRP channel modulators, and have significantly contributed to our current knowledge of TRP biology.

TRPs have been identified as crucial in the development and progression of cancer. To date, these channels have been grouped into seven subfamilies. Despite their permeability to different ions, the majority of TRPs are more selective to calcium, the most important second messenger implicated in cancer progression. Some TRPs are deregulated in many types of cancer, despite research revealing that TRPs have many different roles that need clarification. The aim of this Special Issue of *Biomolecules*, “Transient Receptor Potential (TRP) Channels: Markers and Therapeutic Targets for Cancer?”, is to provide a broad and updated overview of the main roles of specific TRPs in cancer development and progression. Six manuscripts have been published, namely five reviews and one original research article which, together, encompass several areas of cancer biology.

In their review, Kärki T. and Tojkander S. [1] describe the involvement of TRPV channels as sensors of various extracellular cues. Indeed, by sensing “force-from-lipids” or “force-from-filament”, TRPV2 and TRPV4 channels can directly respond to membrane stress/stretch/tension, or feel the force via interactions with cytoskeletal/structural/adhesive components. This leads to conformational changes and gating of the channel, to subsequently trigger specific cation-dependent intracellular signaling pathways. Epithelial–mesenchymal transition and stiffness of the microenvironment, matrix degradation, and angiogenesis have been considered.

Borgström A. et al. [2] provide a detailed account of the TRPM4 mechanism of action in cancer and discuss new small-molecule TRPM4 inhibitors, given that in different cancers, TRPM4 expression levels are increased. Prostate, colorectal, cervical, endometrial, and breast cancers are the main types analyzed. Moreover, among the anticancer drugs, the authors highlight a TRPM4-specific antibody named M4P, which is found to inhibit TRPM4’s current by binding close to the channel pore.

In their review, Abrahamian C and Grimm C. describe the roles of the two-pore channel and TRPML in melanocytes and melanoma [3]. In particular, TRPML1 seems to be required in melanoma cells to negatively regulate the MAPK pathway and mTORC1 signaling. Consequently, cellular proliferation is promoted by mTORC1, and the MAPK pathway controls the microphthalmia-associated transcription factor (MITF), which is the major regulator of melanoma proliferation and progression.

In the review article by Maggi F. and collaborators [4] the most recent findings on TRP channels in leukemia and lymphoma malignances are addressed. In acute myeloid leukemia, TRPM2 seems to play a leading role in influencing cancer cell survival, doxorubicin sensitivity, and myeloid differentiation. TRPV2, TRPM7, and TRPC1 are the main channels involved in chronic myeloid leukemia. The silencing of TRPV2 induces apoptotic cell death in the K562 line, and the blockage or alteration of TRPM7 results in a reduction in K562 proliferation. Moreover, TRPV1 is related to SOCE activity. Moving to the subject of acute lymphoblastic leukemia, TRPV1 activation induces apoptosis in both Jurkat cells and cell-derived T-cell acute lymphoblastic leukemia patients. TRPV6 is essential for Jurkat cell migration and oncogenic activity through lipid raft integrity. TRPM2 is crucial in Jurkat cell-cycle regulation. Regarding chronic lymphocytic leukemia, TRPC1 is involved in the production of anti-inflammatory cytokines, which promote cell survival. Moreover, many in vitro studies performed in patient-derived cells demonstrate the ability of TRP-targeting therapy to inhibit cell proliferation and improve the effects of traditional chemotherapy in hematological malignancies, making this protein family a promising target.

Remaining on the topic of hematological malignances, Santoni G. and coworkers [5] evaluated the role of TRPML2 in multiple myeloma cell lines treated with a combination of ibrutinib and bortezomib. In particular, they linked the ibrutinib-resistant U266 and the ibrutinib-sensitive RPMI with a different TRPML2 expression. Ibrutinib-resistant U266 cells are characterized by low TRPML2 expression, and RNA-silencing experiments in RPMI cells confirmed the TRPML2 dependence of the ibrutinib response. Additionally, the combination of ibrutinib with bortezomib confirms the central role of TRPML2. Thus, this manuscript underlines the importance of investigating TRPML2 expression for better stratification of ibrutinib sensitivity in MM patients. 

A contribution by Duitama M. et al. [6] summarizes TRP involvement in cancer pain. Indeed, these channels are also critical receptors in the transduction of nociceptive stimuli. The tumor microenvironment provides mediators that may play an important role in direct activation or sensitization, by lowering the activation threshold of their TRPV1 and TRPA1 channels, which are involved in pain response. Thus, this review discusses the pathways involved in the role of TRPV1 and TRPA1 in cancer pain, and the effects of specific agonists or antagonists to desensitize them, as a strategy to promote an analgesic effect.

To conclude, this Special Issue of Biomolecules describes important findings related to the role of TRP channels in cancer, highlighting several mechanisms in which they are involved. These data can be extremely useful in providing new targets, and in developing pharmacological strategies aimed at modulating their activity for therapeutic and clinical purposes.
